# Soluble receptor activator of nuclear factor κB ligand/osteoprotegerin ratio is increased in systemic lupus erythematosus patients

**DOI:** 10.1186/ar3500

**Published:** 2011-10-25

**Authors:** Diana Carmona-Fernandes, Maria José Santos, Inês Pedro Perpétuo, João Eurico Fonseca, Helena Canhão

**Affiliations:** 1 Rheumatology Research Unit, Instituto de Medicina Molecular, Faculdade de Medicina da Universidade de Lisboa, Edifício Egas Moniz, Av. Prof. Egas Moniz, 1649-028 Lisboa, Portugal; 2 Rheumatology Department, Hospital Garcia de Orta, Av. Torrado da Silva, 2801-951 Almada, Portugal; 3 Rheumatology and Bone Metabolic Diseases Department, Hospital de Santa Maria, Av. Prof. Egas Moniz, 1649-035 Lisboa, Portugal

**Keywords:** sRANKL, osteoprotegerin, systemic lupus erythematosus, osteoclastogenesis

## Abstract

**Introduction:**

Systemic lupus erythematosus (SLE) patients have lower bone mineral density and increased fracture risk when compared with healthy individuals, due to distinct factors and mechanisms. Bone remodeling is a tightly orchestrated process dependent on several factors, including the balance between receptor activator of nuclear factor κB ligand (RANKL) and osteoprotegerin (OPG).

Our aim was to assess serum OPG and soluble RANKL (sRANKL) levels as well as sRANKL/OPG ratio in female SLE patients and compare it with female controls.

**Methods:**

We have evaluated 103 SLE patients and 114 healthy controls, all Caucasian females. All participants underwent a clinical and laboratory evaluation. sRANKL and OPG were quantified in serum by ELISA based methods. sRANKL, OPG and sRANKL/OPG ratio levels were compared between SLE patients and age, sex and race matched healthy controls. For SLE patients, a multivariate analysis was performed, to find the possible predictors of the changes in sRANKL, OPG and sRANKL/OPG ratio levels.

**Results:**

Although sRANKL levels did not differ between the two groups, serum OPG was lower in SLE patients (*P *< 0.001). This led to an increased sRANKL/OPG ratio (*P *= 0.010) in the patients' group.

The multivariate analysis was performed considering age and other clinical and laboratorial potential confounders for these variations in the SLE patients group. We have showed that age (*P *= 0.001) and levels of anti-Sm antibodies (*P *= 0.016) were independent predictors of sRANKL/OPG ratio variations in SLE patients. No relationship with therapy or disease activity measured by SLEDAI2K was found.

**Conclusions:**

These results are suggestive of increased osteoclastic stimuli driven by the SLE disease mechanisms.

## Introduction

Systemic lupus erythematosus (SLE) is a chronic, multisystemic disease of unknown etiology characterized by chronic inflammation and damage to various organs and systems due to the production of autoreactive cells and antibodies [1-3].

SLE patients have lower bone mineral density (BMD) when compared with healthy individuals and are at increased risk of fracture [4-7]. Although corticosteroid exposure is a major contributor to bone loss in SLE [4,5,8]. disease activity and associated co-morbidities may contribute to this process [5,8]. In addition, vitamin D deficiency is a common finding among SLE patients, further contributing to impaired bone health [5].

Bone remodeling is a tightly orchestrated process in which osteoclasts attach to the bone surface and remove bone. After resorption, osteoblasts migrate into the lacunae and produce new bone, which then mineralizes [3,9]. Expression of the receptor activator of nuclear factor κB ligand (RANKL) by osteoblasts is essential to osteoclastogenesis. Osteoprotegerin (OPG) is a soluble receptor for RANKL that prevents RANK/RANKL interaction. Therefore, the RANKL/OPG ratio is critical for the control of bone resorption [10-12]. Increased RANKL/OPG ratio has been described in autoimmune diseases, such as rheumatoid arthritis, and was associated with an increased bone loss [7,13,14]. Taking these arguments into consideration, we have hypothesized that a RANKL/OPG imbalance is also present in SLE patients.

In the present work we aimed to assess the RANKL/OPG balance in SLE patients by quantifying serum OPG and sRANKL levels and their ratio in SLE patients and healthy controls. Additionally, in SLE patients we have looked at predictors of serum levels of these proteins and having as covariates disease features, co-morbidities and medications.

## Materials and methods

### Patients

Consecutive Portuguese Caucasian SLE women were recruited from the rheumatology outpatient clinics of Hospital de Santa Maria, Lisbon and Hospital Garcia de Orta, Almada, Portugal. All enrolled patients fulfilled the classification criteria of the American College of Rheumatology for SLE (1997) and had normal renal function defined as serum creatinine < 1.5 mg/dl. A control group matched to age, sex and race was also recruited, and was composed of healthy Caucasian female volunteers, who had not been diagnosed with SLE, nor had any inflammatory or bone disease and were not receiving corticosteroids or other medications known to interfere with bone metabolism. In this study, 103 SLE patients and 114 healthy controls were enrolled. This study was approved by the local Ethics Committees and all participants signed a written informed consent.

All participants underwent a standardized clinical and laboratory evaluation [15]. Information about age, height, weight, body mass index (BMI), smoking habits, alcohol intake, menopause, co-morbidities (hypertension, hyperlipidemia, diabetes mellitus, hypo or hyperthyroidism) and medication was collected.

For SLE patients, information considering age at disease diagnosis, disease duration, cumulative clinical manifestations, presence of autoantibodies, current disease activity (evaluated using the Systemic Lupus Erythematosus Disease Activity Index 2000 (SLEDAI2K) [16]), and cumulative damage (scored in accordance to the Systemic Lupus International Collaborating Clinics/ACR Damage Index (SLICC) [17]) were also obtained.

### Laboratorial determinations

A blood sample was collected from all subjects for measurement of erythrocyte sedimentation rate (ESR), C-reactive protein (CRP), lipids (total cholesterol, HDL, LDL and tryglicerides), and anti-dsDNA and anti-Sm antibody titers.

Serum was obtained by blood centrifugation at 1,250 g, 10', at 4°C and then preserved at -80°C until use for sRANKL and OPG quantification.

sRANKL quantification was performed using the ampli sRANKL human ELISA (Immunodiagnostic Systems, Boldon, UK). OPG was quantified using the Bender MedSystems (Vienna, Austria) bead-based assay for quantitative detection of soluble human analytes by flow cytometry. Both protocols were performed according to the manufacturer's instructions.

### Statistical analysis

For statistical purposes samples undetectable or below the limit of detection (LOD) were considered to have the lower LOD value supplied by the manufacturer. Results were reported as means (standard deviation), medians (interquartile range) for continuous or proportions for categoric variables. sRANKL and OPG levels and sRANKL/OPG ratio were compared between SLE patients and healthy controls groups using the non-parametric Mann-Whitney test.

Subsequently, the impact of demographic parameters, clinical features, therapeutics and disease characteristics on these proteins and their ratio was investigated for SLE patients using univariate followed by multivariate linear regression analyses. All variables related to the studied outcome in univariate analyses at a *P*-value < 0.05 were considered potential predictors and entered in multivariate linear regression models. The selection of covariates was stepwise by backward selection, according to the level of significance. Before performing regression analysis, sRANKL, OPG, and sRANKL/OPG ratio were logarithmically transformed for approximation to normality and to approximate the residuals to the normality in multiple linear regressions.

Statistical calculations were performed using Statistical Package for the Social Sciences (SPSS) Statistics Software, v.15.0 (SPSS Inc., Chicago, USA) and a two-tailed *P*-value < 0.05 was selected as significant.

## Results

A total of 103 SLE patients and 114 healthy controls with comparable baseline demographic characteristics and co-morbidities were studied (Table 1). SLE patients had a mean age at disease diagnosis of 35.6 ± 14.4 (range 9.0 to 80.0) years, 8.2 ± 6.6 (range 0.2 to 34.2) years of disease duration, a mean SLEDAI2K of 3.5 ± 4.5 (range 0 to 21) and a SLICC damage score of 0.8 ± 1.4 (range 0 to 8). A total of 60.2% of the patients were currently receiving corticosteroids in a mean daily dose of 12.7 mg.

**Table 1:** SLE patients and healthy controls characteristics

	SLE patients	Healthy controls	*P-*value
n	103	114	**-**
Age, years	44.9 ± 14.1	47.4 ± 13.5	0.182
BMI, Kg/m^2^	26.6 ± 4.9	26.8 ± 4.9	0.689
Current smokers, n (%)	14 (15.6%)	23 (22.1%)	0.246
Alcohol intake, n (%)	4 (3.9%)	4 (3.6%)	0.602
Postmenopausal, n (%)	44 (49.4%)	57 (53.3%)	0.593
Arterial hypertension, n (%)	52 (50.5%)	47 (42.7%)	0.257
Hyperlipidemia, n (%)	66 (64.1%)	66 (60.6%)	0.596
Diabetes mellitus, n (%)	5 (4.9%)	8 (7.2%)	0.472

Values represent mean ± standard deviation or frequencies of the individuals that presented the characteristic. Differences were assessed using T-test for continuous variables or χ^2 ^or Fisher's exact test for proportions.

BMI, body mass index; SLE, Systemic Lupus Erythematosus

No significant differences were found between the two groups regarding sRANKL concentration. However, a statistically significant lower value was found for OPG levels in SLE patients (*P *< 0.001) compared to healthy controls. Consequently, an increase in sRANKL/OPG ratio (*P *= 0.010) was found in SLE patients compared to the healthy control group (Table 2).

**Table 2:** sRANKL, OPG and sRANKL/OPG ratio levels in SLE patients and healthy controls

	SLE patients	Healthy controls	*P-*value
sRANKL	0.40 (0.02 to 25.99)	0.40 (0.29 to 67.64)	0.372
OPG	69.02 (17.42 to 500.90)	95.14 (1.56 to 1069.13)	< 0.001
sRANKL/OPG ratio	0.0103 (0.001 to 0.817)	0.0056 (0.000 to 1.866)	0.010

Values represent median (IQR). Differences were assessed by non-parametric Mann-Whitney *U *test.

In 49.5% of SLE patients and 63.2% of healthy controls, the sRANKL levels were below the limit of detection (LOD) of the quantification method. Regarding OPG quantification, there were no samples with concentrations below the LOD.

IQR, interquartile range; OPG, osteoprotegerin; SLE, Systemic Lupus Erythematosus; sRANKL, soluble RANKL

The adjusted relationship between demographic parameters, clinical features, therapies and disease characteristics with sRANKL, OPG and sRANKL/OPG ratio was further analyzed in SLE patients (Table 3).

**Table 3:** Predictors of sRANKL, OPG and sRANKL/OPG ratio levels in SLE patients (after multivariate analysis)

Possible predictors	log(sRANKL)		log(OPG)		log(sRANKL/OPG ratio)	
	**Univariate analysis**	**Multivariate analysis^‡^**	**Univariate analysis**	**Multivariate analysis^§^**	**Univariate analysis**	**Multivariate** analysis*
	**β coefficient (95% CI)**	**β coefficient** **(95% CI)**	**β coefficient (95% CI)**	**β coefficient (95% CI)**	**β coefficient (95% CI)**	**β coefficient** **(95% CI)**
	***P-*value**	***P-*value**	***P-*value**	***P-*value**	***P-*value**	***P-*value**
Age	-0.304 (-0.049 to -0.012)	-0.232 (-0.043 to -0.004)	0.218 (0.001 to 0.015)		-0.363 (-0.058 to -0.019)	-0.326 (-0.065 to -0.015)
	0.002	0.017	0.027		< 0.001	0.001
BMI	-0.249 (-0.126 to -0.016)				-0.211 (-0.123 to -0.005)	
	0.011				0.033	
Menopausal status	-0.210 (-1.131 to -0.006)		0.211 (0.003 to 0.439)		-0.274 (-1.379 to -0.200)	
	0.048		0.047		0.009	
Lipid lowering therapy					-0.214 (-1.451 to -0.068)	
					0.032	
Antihypertensive therapy			0.217 (0.026 to 0.438) 0.028			
Diabetes mellitus			0.271 (0.198 to 1.122)	0.247 (0.117 to 0.984)		
			0.006	0.013		
Triglycerides			0.266 (0.000 to 0.003)	0.306 (0.001 to 0.004)		
			0.011	0.02		
Age at disease onset	-0.268 (-0.045 to -0.008)		0.237 (0.002 to 0.016)		-0.335 (-0.054 to -0.016)	
	0.006		0.016		0.001	
Malar rash	0.251 (0.166 to 1.246)	0.243 (0.150 to 1.230)			0.201 (0.019 to 1.178)	
	0.011	0.013			0.043	
Arthritis			-0.272 (-0.556 to -0.098)			
			0.005			
Pleuritis					-0.197 (-1.555 to -0.010)	
					0.047	
Anti-Sm titers	0.224 (0.003 to 0.051)	0.227 (0.005 to 0.051)			0.264 (0.009 to 0.059)	0.229 (0.006 to 0.053)
	0.026	0.018			0.008	0.016
Anti-dsDNA titers	0.204 (0.000 to 0.007)		-0.208 (-0.003 to 0.000)	-0.239 (-0.004 to 0.000)	0.268 (0.001 to 0.009)	
	0.047		0.043	0.016	0.009	

Multivariate analysis results from multiple linear regression analysis. The total explained variance of the model is (‡) R^2 ^= 0.185, (§) R^2 ^= 0.237, and (*) R^2 ^= 0.175.

OPG, osteoprotegerin; SLE, Systemic Lupus Erythematosus; sRANKL, soluble RANKL

In univariate analysis, age, BMI, menopausal status, age at disease onset, the presence of malar rash, and anti-Sm and anti-dsDNA antibody quantifications were significantly associated with sRANKL levels. These possible predictors were included in a multivariate analysis and age (β = -0.232, 95% CI -0.043 to -0.004; *P *= 0.017), malar rash (β = 0.243, 95% CI 0.150 to 1.230; *P *= 0.013), and levels of anti-Sm antibodies (β = 0.227, 95% CI 0.005 to 0.051; *P *= 0.018) were identified as independent predictors of sRANKL levels in SLE patients.

The variables associated with OPG levels in univariate analysis were age, menopausal status, antihypertensive therapy, diabetes mellitus, triglycerides, age at disease onset, arthritis and anti-dsDNA titers. All these potential predictors were included in a multivariate analysis. Diabetes mellitus (β = 0.247, 95% CI 0.117 to 0.984; *P *= 0.013), anti-dsDNA levels (β = -0.239, 95% CI -.004 to 0.000; *P *= 0.016), and triglycerides (β = 0.306, 95% CI 0.001 to 0.004; *P *= 0.002) were found to be independent predictors of OPG levels in the serum of SLE patients.

The same reasoning was applied to the analysis of the sRANKL/OPG ratio in SLE patients. In the univariate analysis age, BMI, menopausal status, lipid-lowering therapy, age at disease onset, the presence of malar rash, pleuritis and the levels of anti-dsDNA and anti-Sm antibodies came out as possible predictors for changes in the ratio values. After multivariate analysis age (β = -0.326, 95% CI -0.055 to -0.015; *P *= 0.001) and levels of anti-Sm antibodies (β = 0.229, 95% CI 0.006 to 0.053; *P *= 0.016) were independently associated with sRANKL/OPG ratio levels in SLE patients.

We found no relationship between sRANKL/OPG ratio and the inflammatory parameters ESR and CRP. In addition, we found no association between the ratio and concomitant medications, such as methotrexate, cyclophosphamide, mycophenolate mofetil or azathioprine. Furthermore, there was also no correlation with corticosteroids (use or actual dose) or disease activity measured by the SLEDAI2K with this ratio.

Although the studied SLE patients presented a wide range of disease duration, this variable did not come out as a predictor of sRANKL, OPG or SRANKL/OPG levels.

## Discussion

The present work provides evidence of increased pro-osteoclastogenic stimuli in SLE women as a result of decreased serum OPG levels and increased sRANKL/OPG ratio.

OPG serum levels were lower in SLE patients than in controls and these levels were negatively associated with anti-dsDNA levels, independently from the contribution of multiple confounders. Raised anti-dsDNA levels are associated with active disease, suggesting that patients with active SLE might be more exposed to the effect of RANKL/RANK interaction as a consequence of diminished OPG levels. We have also found a positive association between serum OPG levels and diabetes mellitus, which is in accordance with previous results [18,19]. Gannagé-Yared and colleagues found an inverse correlation between OPG and triglycerides levels, in a nondiabetic, elderly Lebanese male population [20]. Although in a different population, this relation was not confirmed by our study, since we have found a positive relation between serum OPG and triglycerides.

Serum OPG levels have been scarcely analyzed in the context of SLE. There is a single study reporting higher serum OPG levels in SLE patients, and this relation is even greater in patients with antiphospholipid syndrome, as OPG levels were related to the presence of antiphospholipid antibodies [21]. This relation between OPG and these antibodies was not confirmed by us (data not shown).

On the other hand, urinary OPG levels have been described to be raised in lupus nephritis and correlated with renal disease activity and anti-dsDNA levels [22,23]. However, at this moment it is not clear how these results can be compared with ours as the relationship between serum and urinary OPG levels is unknown.

We have found sRANKL levels to be similar between SLE and healthy control women, but the sRANKL/OPG ratio was increased in SLE patients as compared to controls at the cost of elevated serum OPG levels in SLE. Interestingly, malar rash and elevated levels of anti-Sm autoantibodies, often present in active disease, were associated with sRANKL serum levels. Moreover, in multivariate analysis levels of anti-Sm antibodies were positively associated with sRANKL/OPG ratio. Studies based on sRANKL are sometimes limited by the high percentage of patients that have undetectable circulating levels, due to the fact that the majority of RANKL is membrane bound. Nevertheless, half of our patients had detectable levels of sRANKL and, importantly, it was possible to determine serum OPG levels in all individuals.

Despite the fact that there are no previous references in the literature to the possible effect of SLE on sRANKL and sRANKL/OPG ratio, an imbalance of this ratio has been described in autoimmune diseases, such as rheumatoid arthritis [13,24]. This finding may be of clinical relevance as the increase of the sRANKL/OPG ratio has been related with increased bone loss in immune mediated inflammatory diseases [12,25]. The independent association of the sRANKL/OPG ratio with anti-Sm autoantibodies and the absence of association with corticosteroid use or dose are particularly relevant, as they are suggestive that SLE *per se *might be important in accelerating osteoclastogenesis and consequently, bone loss.

## Conclusions

In summary, we have shown reduced OPG levels and consequently a raised sRANKL/OPG ratio in female SLE patients as compared to healthy controls. An association between anti-dsDNA and OPG levels and between anti-Sm and sRANKL levels and sRANKL/OPG ratio were also observed in SLE patients. Taken together, these observations are suggestive of increased osteoclastic stimuli driven by SLE disease mechanisms.

## Abbreviations

Anti-dsDNA: anti-double stranded DNA; Anti-Sm: anti-Smith; BMD: bone mineral density; BMI: body mass index; CRP: C-reactive protein; EDTA: ethylenediamine tetraacetic acid; ELISA: enzyme linked immunosorbent assay; ESR: erythrocyte sedimentation rate; HDL: high-density lipoprotein; LDL: low-density lipoprotein; LOD: limit of detection; OPG: osteoprotegerin; SLE: systemic lupus erythematosus; RANK: receptor activator of nuclear factor κB; RANKL: receptor activator of nuclear factor κB ligand; sRANKL: soluble RANKL; SLEDAI2K: SLE disease activity index; SLICC: systemic lupus international collaborating clinics/ACR damage index; SPSS: statistical package for the social sciences.

## Competing interests

The authors declare that they have no competing interests.

## Authors' contributions

DCF carried out laboratorial protein determinations and participated in the design of the study, statistical analysis and manuscript elaboration. MJS performed clinical evaluation of the patients and participated in the design of study, statistical analysis and manuscript elaboration. IPP helped on laboratorial protein determinations and on manuscript revision. JEF participated in the design of the study and on manuscript revision. HC participated in the design of the study and on manuscript and statistical analysis revision. All authors read and approved the final manuscript for publication.
